# Binding of the Dual-Action Anti-Parkinsonian Drug AG-0029 to Dopamine D_2_ and Histamine H_3_ Receptors: A PET Study in Healthy Rats

**DOI:** 10.1021/acs.molpharmaceut.2c00121

**Published:** 2022-06-22

**Authors:** Nafiseh Ghazanfari, Aren van Waarde, Janine Doorduin, Jürgen W. A. Sijbesma, Maria Kominia, Martin Koelewijn, Khaled Attia, David Vállez-García, Antoon T. M. Willemsen, André Heeres, Rudi A. J. O. Dierckx, Ton J. Visser, Erik F. J. de Vries, Philip H. Elsinga

**Affiliations:** † Department of Nuclear Medicine and Molecular Imaging, University Medical Center Groningen, University of Groningen, Hanzeplein 1, 9713 GZ Groningen, The Netherlands; ‡ Symeres B.V., Kadijk 3, 9747 AT Groningen, The Netherlands

**Keywords:** Parkinson’s disease, anti-Parkinson drug, [^11^C]raclopride, [^11^C]GSK-189254, dopamine D_2_ receptor, histamine H_3_ receptor, dual-action pharmaceutical, pharmacokinetic modeling

## Abstract

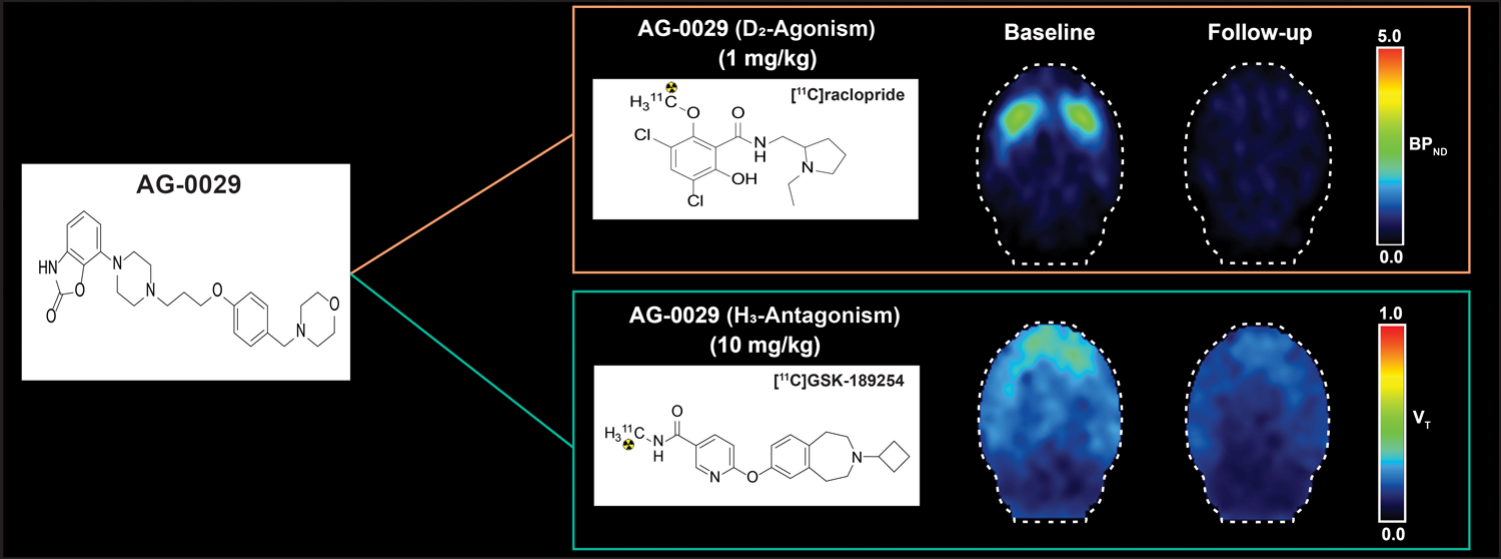

**Introduction**: Parkinson’s disease (PD) is a neurodegenerative disorder characterized by motor dysfunction and a diverse range of nonmotor symptoms. Functional relationships between the dopaminergic and histaminergic systems suggest that dual-action pharmaceuticals like AG-0029 (D_2_/D_3_ agonist/H_3_ antagonist) could ameliorate both the motor and cognitive symptoms of PD. The current study aimed to demonstrate the interaction of AG-0029 with its intended targets in the mammalian brain using positron emission tomography (PET). **Methods**: Healthy male Wistar rats were scanned with a small-animal PET camera, using either the dopamine D_2_/D_3_ receptor ligand [^11^C]raclopride or the histamine H_3_ receptor ligand [^11^C]GSK-189254, before and after treatment with an intravenous, acute, single dose of AG-0029. Dynamic [^11^C]raclopride PET data (60 min duration) were analyzed using the simplified reference tissue model 2 (SRTM2) with cerebellum as reference tissue and the nondisplaceable binding potential as the outcome parameter. Data from dynamic [^11^C]GSK-189254 scans (60 min duration) with arterial blood sampling were analyzed using Logan graphical analysis with the volume of distribution (*V*_T_) as the outcome parameter. Receptor occupancy was estimated using a Lassen plot. **Results**: Dopamine D_2/3_ receptor occupancies in the striatum were 22.6 ± 18.0 and 84.0 ± 3.5% (mean ± SD) after administration of 0.1 and 1 mg/kg AG-0029, respectively. In several brain regions, the *V*_T_ values of [^11^C]GSK-189254 were significantly reduced after pretreatment of rats with 1 or 10 mg/kg AG-0029. The H_3_ receptor occupancies were 11.9 ± 8.5 and 40.3 ± 11.3% for the 1 and 10 mg/kg doses of AG-0029, respectively. **Conclusions**: Target engagement of AG-0029 as an agonist at dopamine D_2_/D_3_ receptors and an antagonist at histamine H_3_ receptors could be demonstrated in the rat brain with [^11^C]raclopride and [^11^C]GSK-189254 PET, respectively. The measured occupancy values reflect the previously reported high (subnanomolar) affinity of AG-0029 to D_2_/D_3_ and moderate (submicromolar) affinity to H_3_ receptors.

## Introduction

Parkinson’s disease (PD) is the second most common age-related neurodegenerative disorder,^1^ associated with neuropathological alterations in the brain. PD is characterized by the pathological accumulation of protein deposits, like α-synuclein and Lewy bodies, that leads to the death of neurons.^2−4^ Not only pathological protein deposition but also changes in neurotransmitter pathways occur years before the onset of clinical manifestations.^5^ In particular, the balance in the nigrostriatal dopamine pathway, which originates in the substantia nigra pars compacta (SNpc) and ends in the striatum, is disturbed in PD patients as a result of significant degeneration of dopaminergic neurons in the SNpc and concomitant dopamine deficiency in the striatum.^6,7^ Postsynaptic striatal dopamine D_2_ receptors are initially upregulated to compensate for the dopamine deficiency,^8^ but are downregulated in advanced stages of the disease.^9,10^

When the dopamine deficiency is too large, it gives rise to motor dysfunction, such as slowing of movement, muscular rigidity, and resting tremor.^11^ PD is also compromising the integrity of other ascending subcortical neurotransmitter pathways, such as the cholinergic, serotonergic, and GABAergic systems, which causes nonmotor symptoms (e.g., impaired cognition, mood disturbance).^5,11,12^ The motor and nonmotor symptoms of PD patients reduce their quality of life during the course of the illness.

The common pharmacotherapy for PD consists of the administration of a precursor of dopamine, such as levodopa (L-DOPA), or a dopamine D_2_ receptor agonist.^13^ These strategies address the dopaminergic deficit, thus providing relief of the motor symptoms. However, the relief of the motor symptoms by such therapy is only temporary and other symptoms of PD do not respond to dopaminergic therapy at all.^14^ More effective treatments may be developed by targeting more than a single neurotransmitter pathway and by considering both the motor and nonmotor complications of PD. Such therapies could be based on the administration of multiple drugs, or a single molecule interacting with multiple targets. Dual or multiple-action pharmaceuticals are considered a novel class of anti-Parkinsonian drugs.^15−18^

In addition to the dopamine D_2_ receptor, the histamine-3 (H_3_) receptor has received particular attention since it acts as a modulator in the release of various neurotransmitters that are involved in the pathophysiology of PD.^6,7,19−22^ There is evidence that the administration of an H_3_ receptor antagonist can potentially ameliorate various symptoms in PD.^13,14,23,24^ Based on preclinical studies in animals and humans, several H_3_ antagonists have been proposed as a treatment for cognitive impairment.^25−35^ This concept could also be important for the treatment of PD, since cognitive impairment is a major nonmotor symptom in PD patients, with a prevalence rate 3 times higher than in the general population.^36−38^

**Figure 1:**
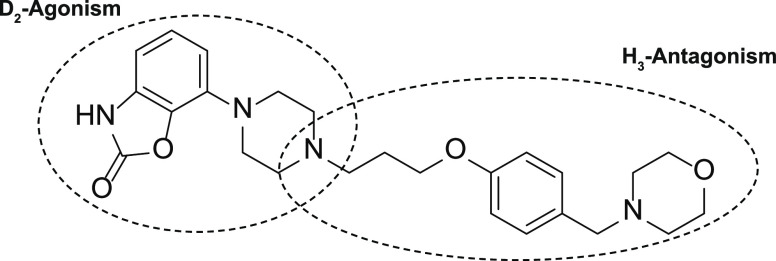
Chemical structure of AG-0029.

As part of a collaborative drug discovery program between Angita Pharmaceuticals, Syncom and Brains Online, 7-(4-(3-(4-(morpholinomethyl) phenoxy)propyl)piperazin-1-yl) benzo[*d*]oxazol-2(3H)-one (AG-0029) was developed (Figure 1). This dual-action drug combines dopamine D_2_ agonism with H_3_ antagonism in an attempt to improve both the motor and nonmotor symptoms of PD. Initial preclinical assessments revealed that AG-0029 is a very potent agonist (EC_50_ 0.08 nM) to dopamine D_2_ receptors and a moderate affinity antagonist (IC_50_ 111 nM) to H_3_ receptors.^39^ These actions of AG-0029 were proven by microdialysis measurements of extracellular dopamine levels in the rat striatum and histamine levels in the rat prefrontal cortex. In 6-hydroxydopamine-lesioned rats, AG-0029 improved motor symptoms (contralateral rotation) and showed a cognition-enhancing effect (novel object recognition test).^40^

To gain more insight into the target engagement of the dual-action drug AG-0029, positron emission tomography (PET) imaging can enable the assessment of receptor availability inside the brain of a living subject at baseline and after administration of a test drug so that each animal acts as its own control.^41^ In the present study, we aimed to quantify the in vivo occupancy of dopamine D_2_/D_3_ and histamine H_3_ receptors by AG-0029 in the brain of healthy rats by PET imaging, using the D_2_/D_3_ receptor ligand [^11^C]raclopride and the H_3_ receptor ligand [^11^C]GSK-189254.

## Materials and Methods

### Tracer Synthesis

[^11^C]Raclopride was produced according to a method described in the literature.^42^ The production of [^11^C]raclopride was accomplished by alkylation of the commercially available precursor S-(+)-O-desmethylraclopride (ABX, Radeberg, Germany). As described in detail by Plisson^43^ and Wang,^44^ the synthesis of [^11^C]GSK-189254 was accomplished via ^11^C-methylation of the amide precursor GSK-185071B with [^11^C]CH_3_I in DMSO at 80 °C for 4 min.

### Animals

In total 22 male Wistar rats (Hsd/Cpb:WU) were obtained from Envigo (Netherlands) at the age of 9 weeks. After arrival, the rats were kept under environmentally controlled conditions (20 ± 2 °C, 50–70% humidity, 12 h light/dark cycle, food and water ad libitum) and acclimated for at least 7 days. Handling and maintaining of rats was in accordance with the guidelines of the University of Groningen. All experiments were approved by the Animal Care and Use Committee of the University of Groningen under license AVD105002015166/AVD1050020198648 and protocol no. IvD 15166-01-004/198648-01-004. The rats were randomly divided into four groups to be scanned either with [^11^C]raclopride (*n* = 5/per either low- or high-dose group) or with [^11^C]GSK-189254 (*n* = 6/per either low- or high-dose group), and to receive either a low or a high dose of AG-0029 before the post-dose scan (0.1 and 1 mg/kg in the case of [^11^C]raclopride scans, 1 and 10 mg/kg in the case of [^11^C]GSK-189254 scans).

For [^11^C]GSK-189254 PET, one baseline scan and one post-dose scan in the low-dose group (1 mg/kg), and two baseline scans and one post-dose scan in the high-dose (10 mg/kg) group were not acquired because of failure of the tracer synthesis or failure of the cannulation of the side branch of the femoral artery. The post-dose scan of one rat from the high-dose (10 mg/kg) group was excluded from the analysis because the AG-0029 drug solution was not adequately administered.

### PET Imaging

All PET scans were made using a MicroPET Focus 220 camera (Siemens Medical Solutions USA, Inc., Malvern, PA). Rats were anesthetized with a mixture of isoflurane and oxygen (5% isoflurane for induction and 1.5–2.5% for maintenance). The body temperature of anesthetized rats was kept close to the normal value by keeping the rats on electronically controlled heating pads (M2M Imaging, Cleveland, OH) during the experiments. Eye salve was applied to prevent dehydration of the cornea. A tail vein was catheterized for injection of the radiotracer. In the group that was scanned with [^11^C]GSK-189254, a second cannula was placed in a side branch of the femoral artery for collection of arterial blood samples, using a previously published procedure that allows longitudinal PET studies with repeated arterial cannulation.^45^ Rats were positioned with the brain in the field of view and, prior to each emission scan, a transmission scan was made using a ^57^Co point source, for attenuation and scatter correction of the final PET images. The body temperature of the rats was continuously recorded, using a rectal PTC thermometer (PicoTechnology, St. Neots, U.K.). The heart rate and oxygen level of the blood were monitored and recorded at 10 min intervals, using a pulse oximeter (PulseSense, Nonin Medical, Plymouth, MN). The rats were first scanned at baseline and 7 days later the post-dose scan was acquired.

Before the start of the [^11^C]raclopride PET scan, saline as the vehicle (control group), 0.1 mg/kg (low dose) or 1 mg/kg (high dose) of AG-0029 in saline was intravenously injected. After ∼35 min, a dose of [^11^C]raclopride (41.2 ± 4.8 MBq at baseline; 39.8 ± 3.3 MBq (0.1 mg/kg) and 41.4 ± 8.1 MBq (1 mg/kg) for post-dose scans) with the injected mass of 1.6 ± 0.6 nmol was intravenously injected over a period of 1 min using an infusion pump. Simultaneous with the tracer injection, a 60 min dynamic [^11^C]raclopride PET acquisition without blood sampling was started. Approximately 18 min before the start of the [^11^C]GSK-189254 PET scan, vehicle (saline) at baseline or AG-0029 in saline at a low dose of 1 mg/kg or a high dose of 10 mg/kg was intravenously administered. A 60 min dynamic PET acquisition with arterial blood sampling was started simultaneously with the injection of the H_3_ receptor ligand [^11^C]GSK-189254 (6.0 ± 11.0 nmol) at a dose of 41.2 ± 4.8, 51.1 ± 28.9, 51.5 ± 29.2 MBq, for the baseline and post-dose (1 mg/kg and 10 mg/kg) scans, respectively. The administrated concentrations and doses of AG-0029 were chosen based on the affinity of the compound to each target, (D_2_ and H_3_ receptors), and the outcome of a few pilot studies that were performed before the start of the PET experiments with [^11^C]raclopride and [^11^C]GSK-189254. After the baseline scans, all rats were allowed to wake up and to recover in their home cage. After the post-dose scans, the rats were terminated by extirpation of the heart under deep anesthesia.

### Arterial Blood Sampling and Metabolite Analysis

During dynamic PET scans with [^11^C]GSK-189254, small arterial blood samples (volume 0.1–0.15 mL) were collected manually at 10, 20, 30, 40, 50, 60, 90 s and 2, 3, 5, 7.5, 10, 15, 30, and 60 min after injection of [^11^C]GSK-189254. Whole blood (25 μL) was collected from each sample, and the remaining sample was centrifuged for 5 min at 13 000*g* (Mikro20, Hettich, Germany) to obtain 25 μL of plasma. The radioactivity in plasma and whole blood was measured with an automated γ counter (Wizard2480, PerkinElmer). To keep the rats alive for the second scan, the total amount of withdrawn blood from each animal was kept below 2 mL, which is less than 10% of the total blood volume in a rat. Thus, only a single extra arterial blood sample with a larger blood volume (0.5 mL) was drawn at 2, 5, 10, 20, 30, 40, or 60 min in baseline scans for metabolite analysis (at different time points in different animals). Two extra samples for metabolite analysis were collected in each animal during post-dose scans, considering the rats were terminated immediately after the post-dose scan. Metabolite data acquired from these large blood samples were used to generate population-based metabolite correction curves for the baseline, 1 and 10 mg/kg scans. After centrifugation of whole blood samples, 0.5 mL acetonitrile was added to plasma and the mixture was vortexed for 1 min. The acetonitrile–plasma mixture was centrifuged for 3 min and the supernatant was passed through a Millex-HV 4-mm syringe filter (pore size 0.45 μm). The filtered supernatant was injected on a semipreparative HPLC column (Gemini C18 110 Å, 250 × 10 mm^2^, 5 μm) that was eluted with a mobile phase of 0.1 M ammonium acetate/acetonitrile (75:25 v/v). Thirty-second fractions of the eluate were collected, and radioactivity in the eluate was measured with a γ-counter (Wizard2480, PerkinElmer). Areas under the parent and metabolite peaks were determined to calculate the fraction of parent [^11^C]GSK-189254 in plasma.

### Image Processing and PET Image Analysis

List mode data from PET emission scans of both tracers were normalized and corrected for decay, scatter, random coincidences, and attenuation. The iterative reconstruction was performed using Fourier rebinning, an OSEM algorithm with 4 iterations, and 16 subsets resulting in 21 time frames composed of 6 × 10, 4 × 30, 2 × 60, 1 × 120, 1 × 180, 4 × 300, and 3 × 600 s of spatial domain information.

The reconstructed [^11^C]raclopride and [^11^C]GSK-189254 images were automatically registered by rigid transformation to specific tracer templates,^46^ which were spatially realigned on a T_2_-weighted MRI scan of the brain of a Wistar rat in Paxinos space (Atlas) to enable image processing and further analysis using PMOD software (version 4.1 PMOD Technologies LLC, Zürich, Switzerland). Striatum and cerebellum were selected for analyzing [^11^C]raclopride data (well-known and validated target and reference regions for this tracer), while for [^11^C]GSK-189254 image analysis 12 brain regions (striatum, cerebellum, parietal cortex, temporal cortex, occipital cortex, frontal cortex, amygdala, hippocampus, hypothalamus, brainstem, midbrain, thalamus) and a VOI covering the whole brain were selected from a rat brain atlas.^46^

### Pharmacokinetic Modeling

For each rat, the mean tissue activity concentrations (kBq/mL) for each time frame were calculated to generate time–activity curves (TACs) for each selected VOI. To facilitate comparison between different rats and scans, the TACs for brain regions, whole blood, and plasma were normalized to standardized uptake values (SUV), based on the formula described below
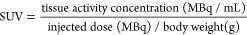
1The nondisplaceable fraction derived binding potential (BP_ND_) and relative delivery influx (*R*_1_) values for [^11^C]raclopride were estimated using the simplified reference tissue model 2 (SRTM2), a well-validated quantification approach for this radioligand.^47^ The reference region clearance constant parameter (*k*′_2_) was determined with the striatum as the target region and then used as the k′_2_ for all other included regions. The BP_ND_ values were estimated using the striatum as the target region with high receptor binding and cerebellum as the reference region with negligible specific uptake.

The TACs of the whole blood and metabolite-corrected arterial plasma during the PET scans were used as input for quantification of the receptor binding of [^11^C]GSK-189254. Based on the results of our previous study,^48^ Logan graphical analysis with a starting equilibrium time of 30 min was selected as the optimal method to estimate the *V*_T_ for each brain region.

### Receptor Occupancy

The receptor occupancy describes the engagement of the test drug with its desired target, with a value of 100% indicating complete receptor occupancy. The BP_ND_ values from the baseline and post-dose [^11^C]raclopride PET scans were used to calculate the receptor occupancy (%) of the D_2/_D_3_ receptor, using the following formula
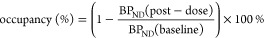
2Since the binding potential of [^11^C]GSK-189254 cannot be reliably estimated, the occupancy of the H_3_ receptor by AG-0029 was estimated from the *V*_T_ of [^11^C]GSK-189254 using a Lassen plot^49^ using the following formula
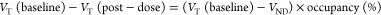
3A graphical representation of this equation, with V_T_(baseline) on the *x*-axis and *V*_T_ (baseline) – *V*_T_ (post-dose) on the *y*-axis, yields a linear relationship with a slope that is equal to the occupancy. It was assumed that the nondisplaceable distribution volume *V*_ND_ was similar for all brain regions.

### Statistical Analysis

Statistical analysis of differences between baseline and post-dose scans was performed using the Generalized Estimating Equations (GEE) model with Bonferroni post hoc analysis^50^ to account for repeated measurements and missing data in the longitudinal study. The independent working correlation matrix in IBM SPSS Statistics (Version 23, Armonk, NY) was selected considering rats and the scan as subject and within-subject variables, respectively. A *p*-value <0.05 was considered to be statistically significant. Results were reported as mean ± standard deviation (SD).

## Results

### Tracer Synthesis

The tracers [^11^C]raclopride and [^11^C]GSK-189254 were obtained in decay-corrected radiochemical yields of 17.0 ± 4.7 and 8.4 ± 6.5% (from [^11^C]CH_4_), with radiochemical purities of 99.2 ± 0.2 and 98.2 ± 1.0% and molar activities of 48.0 ± 34.5 and 33.3 ± 13.4 GBq/μmol, respectively.

### Physiological Effects of AG-0029

**Figure 2:**
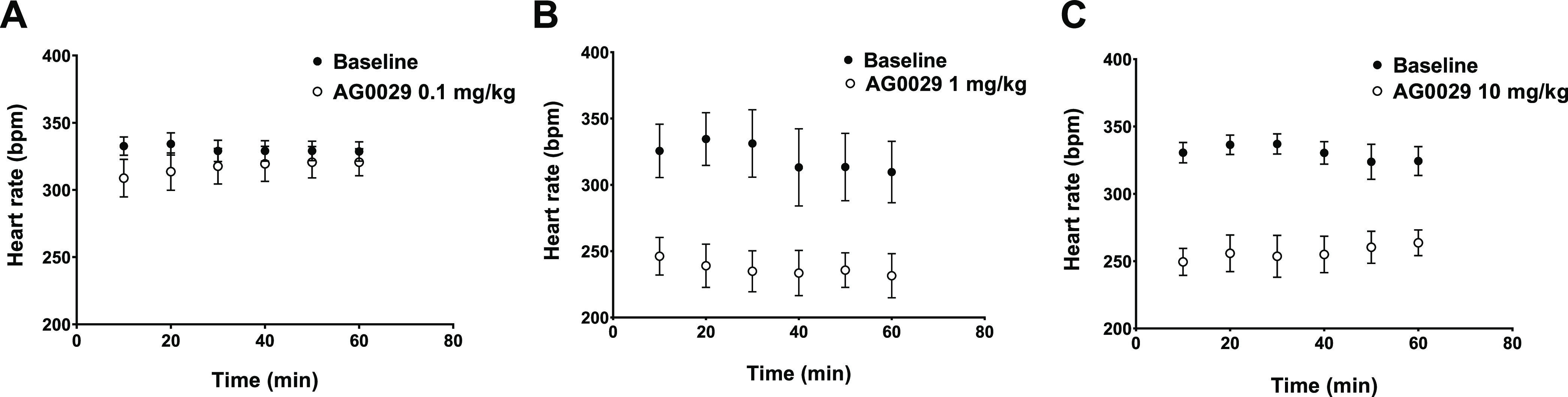
Dose-dependent effect of AG-0029 administration on heart rate.

We did not observe any significant impact of AG-0029 administration (at 0.1, 1, or 10 mg/kg dose) on body temperature. However, administration of 0.1 mg/kg AG-0029 resulted in a transient, minor decrease of heart rate (Figure 2A), and higher doses (1 or 10 mg/kg) caused a significant, pronounced, and sustained bradycardia (Figure 2B,C).

### [^11^C]Raclopride PET to Assess D_2_/D_3_ Receptor Occupancy

**Figure 3:**
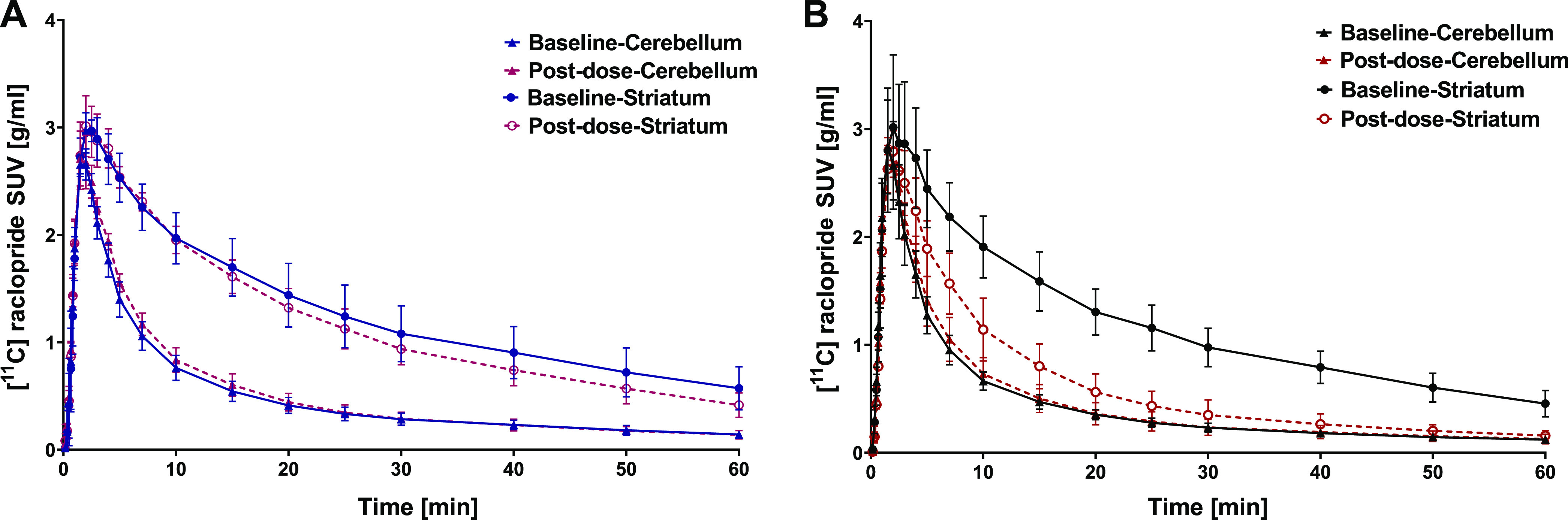
Average [^11^C]raclopride time–activity curves in the striatum (high D_2_ receptor expression) and cerebellum (low D_2_ receptor expression) at baseline and post-dose. Rats received either (A) a low dose (0.1 mg/kg) or (B) a high dose (1 mg/kg) of AG-0029. Data are plotted as mean ± SD.

TACs of [^11^C]raclopride at baseline and after pretreatment with AG-0029 are presented in Figure 3. [^11^C]Raclopride showed the highest uptake in the striatum, the target region with the highest expression of D_2_ receptors. A peak in striatal tracer uptake was observed at 2.2 ± 0.7 min (baseline) after tracer injection, followed by a distinct washout. Administration of 0.1 mg/kg AG-0029 before the scan had little impact on the TACs (Figure 3A). The corresponding areas under the curves (AUC) at baseline and after a dose of 0.1 mg/kg AG-0029 were, respectively, 29.5 ± 4.0 and 31.2 ± 3.2 min × g/mL for the cerebellum (*p* = 0.349) and 76.6 ± 14.2 and 70.3 ± 6.9 min × g/mL for the striatum (*p* = 0.269). The high-dose (1 mg/kg) group presented TACs with AUC values at baseline and post-dose of, respectively, 26.5 ± 3.8 and 28.0 ± 5.3 min × g/mL for the cerebellum (*p* = 0.298) and 71.0 ± 12.3 and 37.6 ± 8.2 min × g/mL for the striatum (*p* < 0.0001) (Figure 3B).

**Figure 4:**
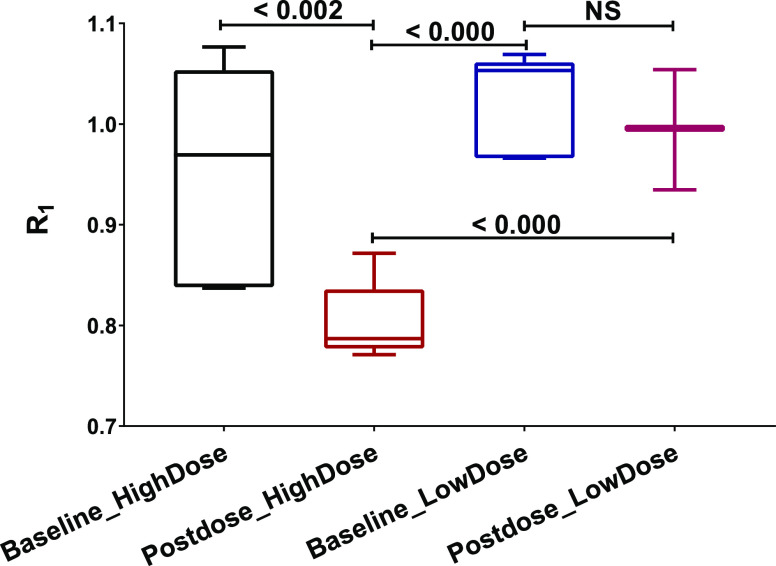
Effect of AG-0029 on the influx (R_1_) of [^11^C]raclopride derived from SRTM2.

There was a statististically significant difference between *R*_1_ derived from SRTM2 at baseline and post-dose in scans with [^11^C]raclopride (Figure 4). The *R*_1_ was reduced from 0.95 ± 0.11 to 0.80 ± 0.04 (mean ± SD) at 1 mg/kg AG-0029 (*p* < 0.002) but was not significantly changed (from 1.02 ± 0.05 to 1.00 ± 0.04, mean ± SD, *p* = NS) at 0.1 mg/kg dose (Figure 4).

**Figure 5:**
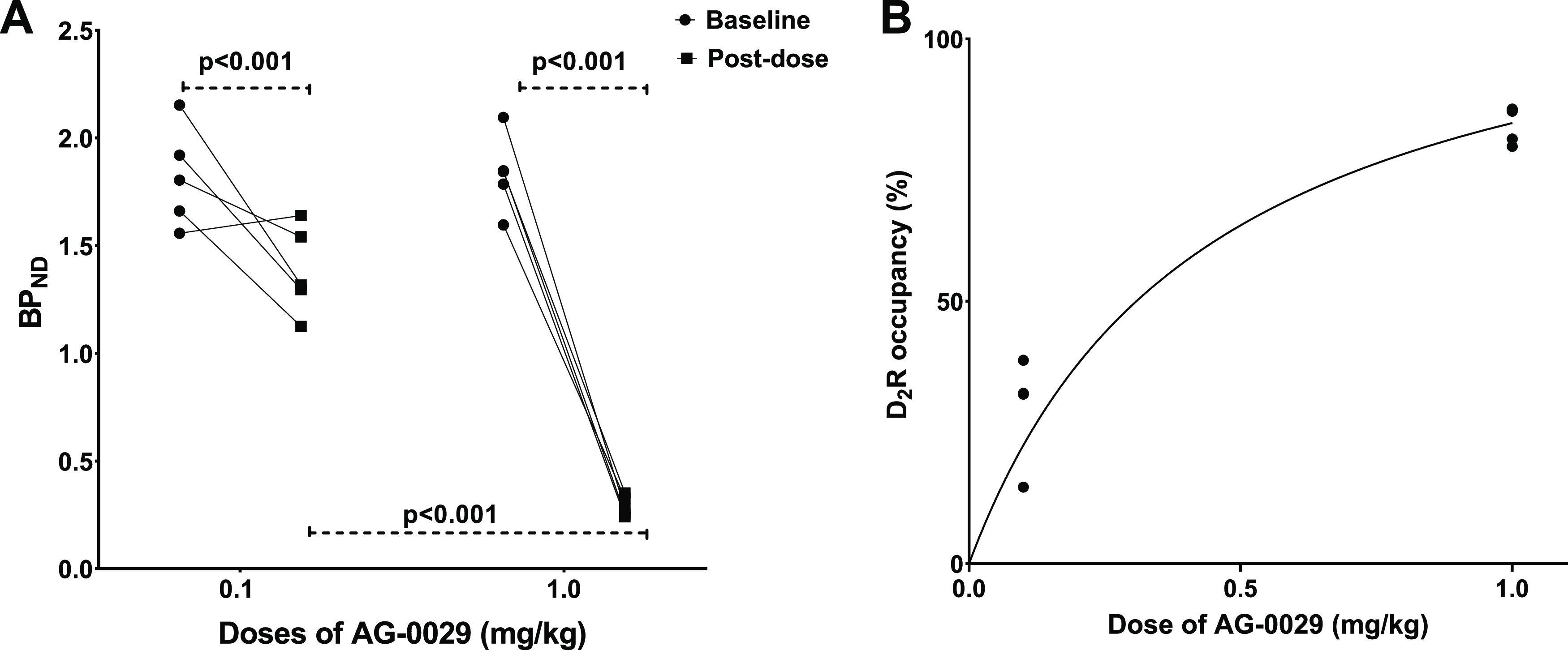
(A) Graphical comparison between baseline and post-dose [^11^C]raclopride binding potential (BP_ND_) in rats pretreated with either 0.1 or 1 mg/kg of AG-0029 as the test drug. (B) Graphical presentation of the striatal D_2_ receptor occupancy versus the AG-0029 dose.

In the striatum, the BP_ND_ values of [^11^C]raclopride were significantly reduced from 1.88 ± 0.21 to 1.32 ± 0.17 (*p* < 0.0001) after a 0.1 mg/kg dose of AG-0029 (*n* = 5) and from 1.82 ± 0.23 to 0.29 ± 0.05 (*p* < 0.0001) after a 1 mg/kg dose of the drug (*n* = 5). For both doses, there was a significant difference in BP_ND_ between the baseline and post-dose scan (Figure 5A). These reductions in BP_ND_ values correspond to a D_2_ receptor occupancy of 29.5 ± 10.4% after a 0.1 mg/kg dose and 84.0 ± 3.5% after a 1 mg/kg dose of AG-0029.

The one-site model can be used to describe the competition between a test drug and a specific receptor ligand for binding to a single class of receptors.^51,52^ This model is based on the equation of Cheng and Prusoff.^53^ A fit of this model to the D_2_ receptor occupancy in the rat striatum (Figure 5B, *R*^2^ = 0.86) indicates that half-maximal receptor occupancy is reached at a dose of 0.26 mg/kg AG-0029, whereas the maximal receptor occupancy (curve maximum) is predicted to be 99.9%.

**Figure 6:**
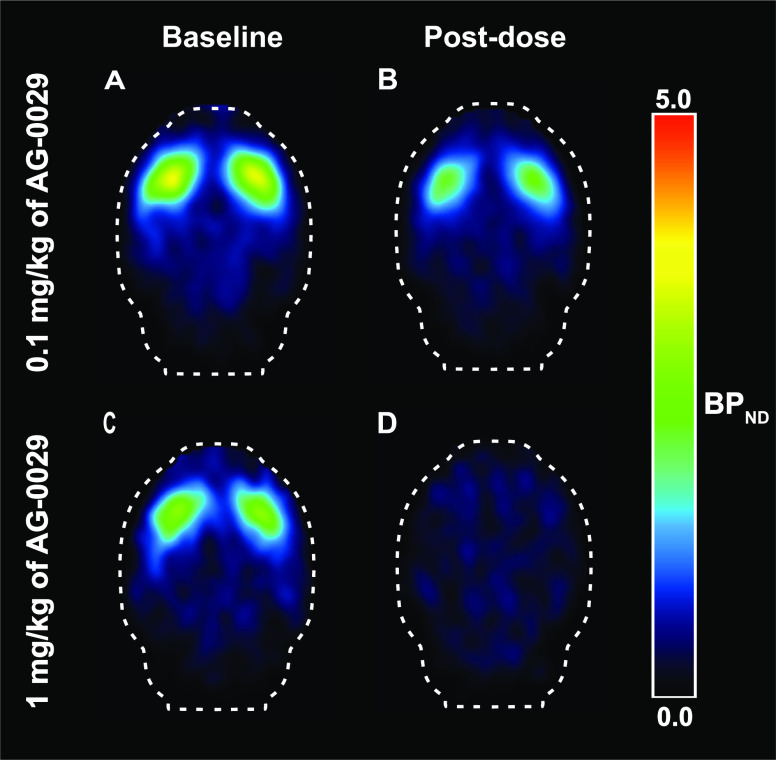
Two sets of BP_ND_ images of [^11^C]raclopride scans acquired at baseline (A, C) and after pretreatment with different doses of AG-0029 (B, D).

A parametric image of the regional binding potential of [^11^C]raclopride in the rat brain is represented in Figure 6.

### Kinetic Modeling of the H_3_ Receptor Ligand [^11^C]GSK-189254

**Figure 7:**
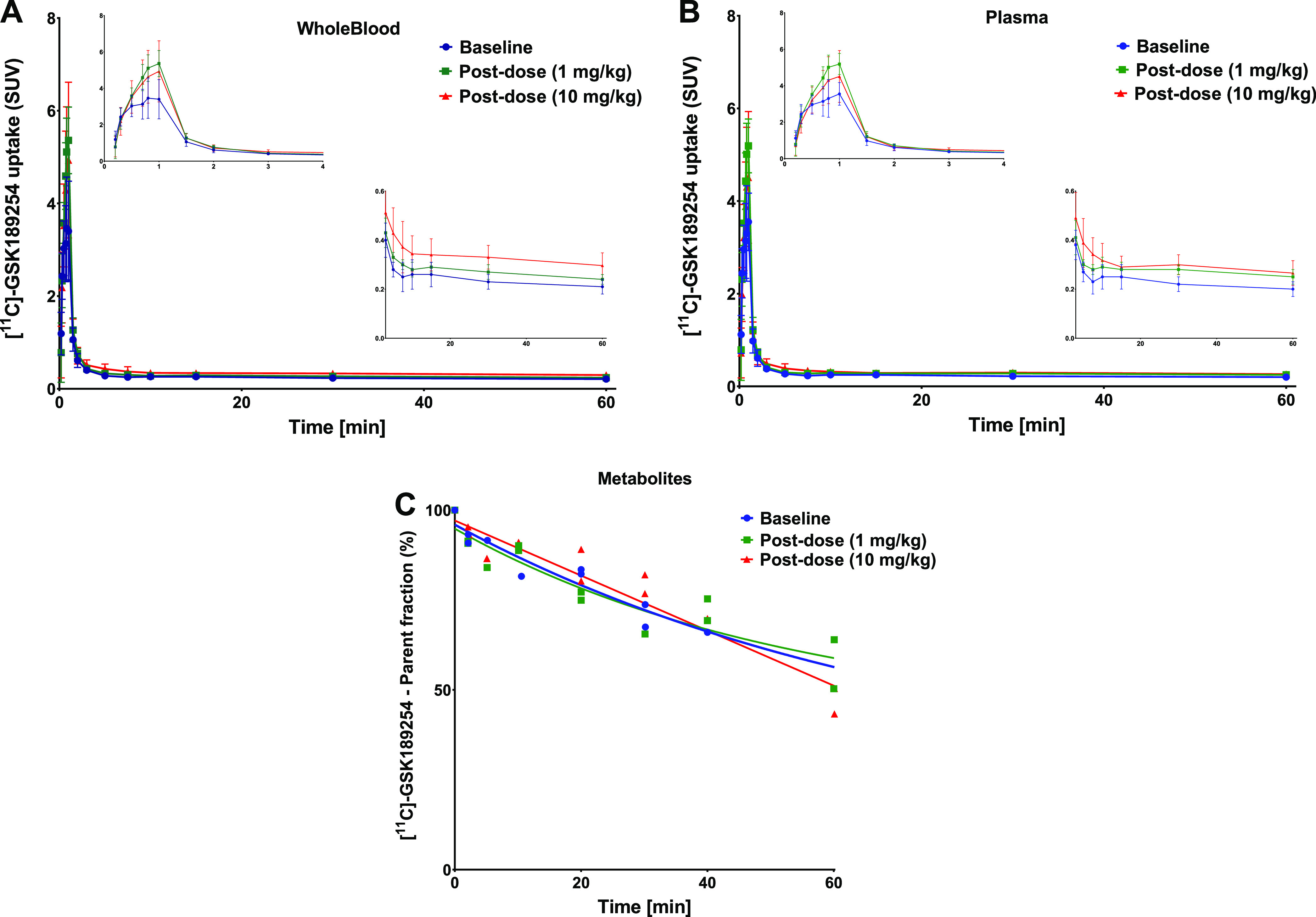
Kinetics of [^11^C]GSK-189254. Time–activity curves in (A) blood and (B) plasma at baseline and after administration of 1 or 10 mg/kg of AG-0029. The insets show the shape of the initial peak. (C) Parent fraction in plasma at baseline and post-dose. Error bars indicate standard deviation.

Blood and plasma TACs, and the time-dependent parent fraction of [^11^C]GSK-189254 in plasma are shown in Figure 7. The peak level of [^11^C]GSK-189254 in blood was observed at ∼1 min after tracer injection with an SUV value of 3.7 ± 0.8 (Figure 7A). Plasma radioactivity reached a maximum SUV of 3.5 ± 0.7.

Administration of 1 mg/kg of the AG-0029 drug increased the activity peak in plasma and blood to an SUV of 4.5 ± 2.2 (*p* = 0.005, compared to the baseline) and 4.6 ± 2.4 (*p* = 0.004), whereas a dose of 10 mg/kg resulted in a similar increase in the peak of activity in plasma and blood with an SUV of 4.0 ± 2.4 (*p* = 0.172) and 4.4 ± 2.7 (*p* = 0.117), respectively. Statistical analysis revealed that administration of AG-0029 did not significantly alter the plasma radioactivity levels (*p* > 0.05) at the end of the 60 min scan.

A one-phase exponential decay function was fitted to the experimental metabolite data of the tracer using a nonlinear regression model to generate a population-based metabolite curve for each drug dose (Figure 7C). These fits indicated metabolic half-lives of 53, 71, and 62 min for the [^11^C]GSK-189254 parent curves at baseline, and after a low dose (1 mg/kg), and high dose (10 mg/kg) of AG-0029. By the end of the 60 min scan, 50 ± 6% of plasma radioactivity still represented intact parent compound, with no significant difference between the 3 AG-0029 dose groups.

**Figure 8:**
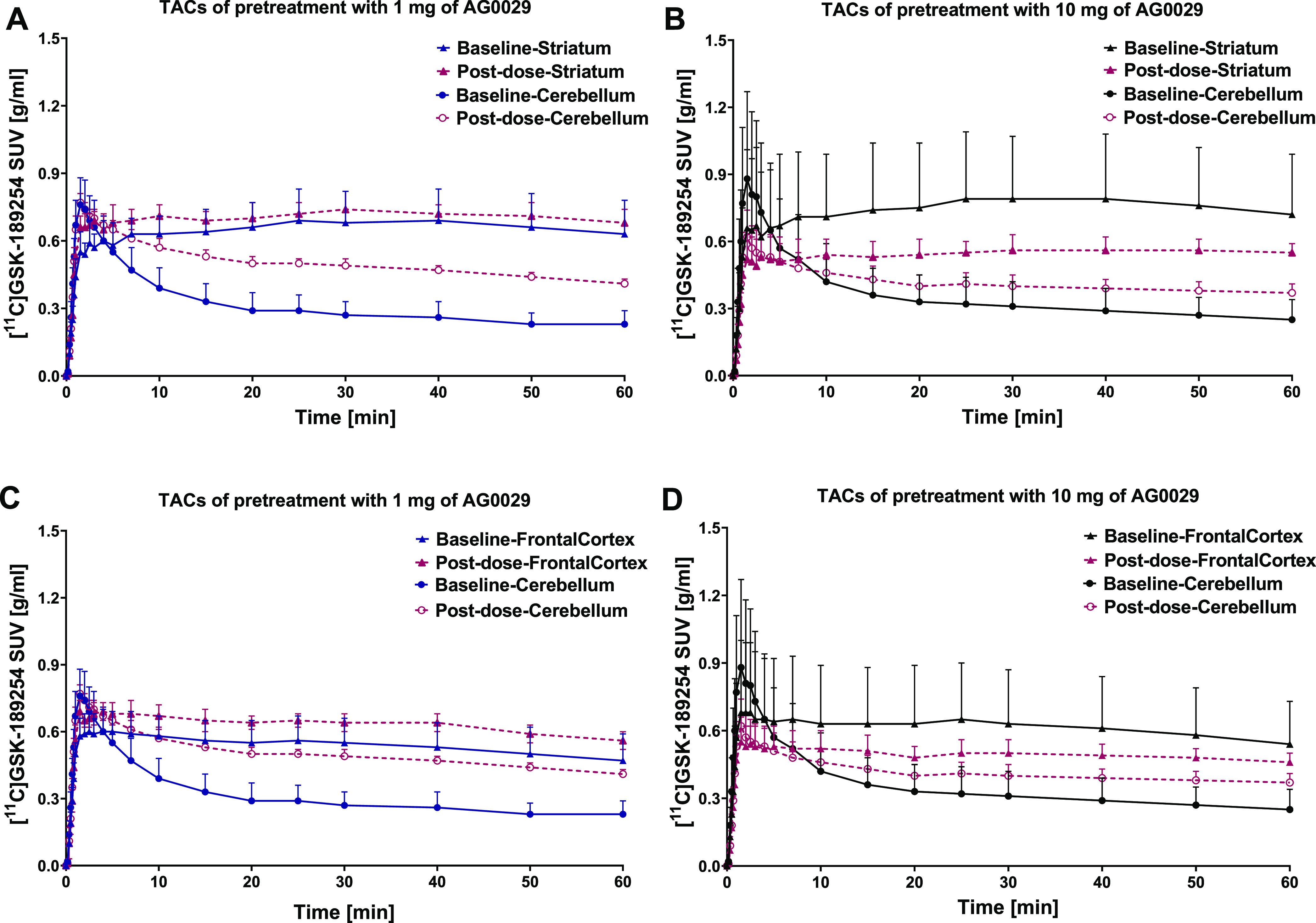
Representative time–activity curves (TACs) of [^11^C]GSK-182954 in the striatum and the cerebellum (A, B) and frontal cortex and the cerebellum (C, D) after pretreatment with a high (10 mg/kg) dose (B, D) and low (1 mg/kg) dose of AG-0029 (A, C).

[^11^C]GSK-189254 showed the highest uptake in the striatum (2.77 ± 0.13), frontal (2.23 ± 0.12) and temporal cortex (2.13 ± 0.13), amygdala (2.10 ± 0.14), and hypothalamus (2.13 ± 0.11), represented as mean and SD.^54^ There was a gradual increase in tracer uptake in regions with high expression of H_3_ receptors, such as the striatum, with the highest uptake being observed ∼25 min after tracer injection. The tracer uptake in the cerebellum, known as a region with a minor expression of H_3_ receptors, was rapid and reached a maximum within 1–2 min after the tracer injection. Peak tracer uptake in the cerebellum was not substantially changed by the drug administration. The perfusion peak was higher in the cerebellum than in H_3_ receptor-rich regions, such as the striatum and frontal cortex. In H_3_ receptor-rich regions, [^11^C]GSK-189254 uptake remained relatively constant over time. After the initial peak, the cerebellum showed a gradual decrease in tracer uptake over time, as shown in Figure 8.

Surprisingly, administration of a low dose (1 mg/kg) of AG-0029 resulted in a small increase in the SUV_50–60_ of H_3_ receptor-rich regions like the striatum (*p* = 0.089) and frontal cortex (*p* = 0.023, statistically significant), but a substantially larger increase in the TAC of the cerebellum (*p* = 0.003, statistically significant), with little H_3_ receptor expression. In contrast, the 10 mg/kg dose of the drug caused a reduction in the TAC of the striatum (*p* = 0.815) and frontal cortex (*p* = 0.840), accompanied with an increase in the TAC of the cerebellum (*p* = 0.821).

*V*_T_ values in the whole-brain and individual brain regions, calculated by Logan analysis, at baseline and after administration of AG-0029 are given in Table 1. The *V*_T_ values were high in brain regions with a known high density of H_3_ receptors like the striatum, frontal cortex, and amygdala. Administration of a 1 mg/kg dose of AG-0029 resulted in significantly lower *V*_T_ values in most brain regions than at baseline, except for the striatum, amygdala, hippocampus, and parietal and temporal cortex. The rats that received the 10 mg/kg dose of AG-0029 showed a significant decrease in *V*_T_ values in all of the brain regions. In addition, statistically significant differences in *V*_T_ between the two administrated doses of AG-0029 were observed in the amygdala (*p* = 0.034), frontal cortex (*p* = 0.002), parietal cortex (*p* = 0.005), striatum (*p* = 0.018), and temporal cortex (*p* = 0.006). There were no significant differences in baseline *V*_T_ values between the low-dose and high-dose groups.

**Table 1:** Regional *V*_T_ Values of [^11^C]GSK-189254 from Logan Graphical Analysis of PET Scans Performed at Baseline or after Administration of 1 and 10 mg/kg of AG-0029^a^

	baseline	post-dose (1 mg/kg)	*p*-values	baseline	post-dose (10 mg/kg)	*p*-values
parietal cortex	2.94 ± 0.32	2.64 ± 0.23	0.054	2.91 ± 0.25	2.03 ± 0.31	<0.001*
temporal cortex	3.26 ± 0.52	2.81 ± 0.26	0.056	3.19 ± 0.24	2.29 ± 0.20	<0.001*
occipital cortex	2.60 ± 0.32	2.20 ± 0.29	0.022*	2.38 ± 0.22	1.85 ± 0.25	0.002*
frontal cortex	3.55 ± 0.40	2.98 ± 0.26	0.003*	3.25 ± 0.23	2.39 ± 0.24	<0.001*
striatum	4.39 ± 0.52	3.84 ± 0.51	0.060	4.16 ± 0.43	2.87 ± 0.43	<0.001*
amygdala	3.10 ± 0.57	2.76 ± 0.48	0.257	3.30 ± 0.32	2.07 ± 0.34	<0.001*
hippocampus	1.62 ± 0.17	1.32 ± 0.18	0.014	1.50 ± 0.11	1.24 ± 0.22	0.003*
hypothalamus	2.71 ± 0.41	2.18 ± 0.35	0.009*	2.44 ± 0.18	1.81 ± 0.36	0.002*
midbrain	3.20 ± 0.30	2.75 ± 0.31	0.001*	3.19 ± 0.29	2.14 ± 0.57	<0.001*
thalamus	1.70 ± 0.19	1.45 ± 0.19	0.021*	1.66 ± 0.18	1.33 ± 0.15	0.002*
cerebellum	2.13 ± 0.21	1.79 ± 0.14	0.002*	2.12 ± 0.20	1.50 ± 0.26	0.013*
brainstem	2.78 ± 0.32	2.37 ± 0.31	0.018*	2.57 ± 0.31	1.90 ± 0.21	0.002*
whole brain	2.68 ± 0.29	2.28 ± 0.24	0.008*	2.56 ± 0.18	1.89 ± 0.23	<0.001*

a Mean ± SD values are listed. *p-values represent the difference between the baseline and post-dose scan.

**Figure 9:**
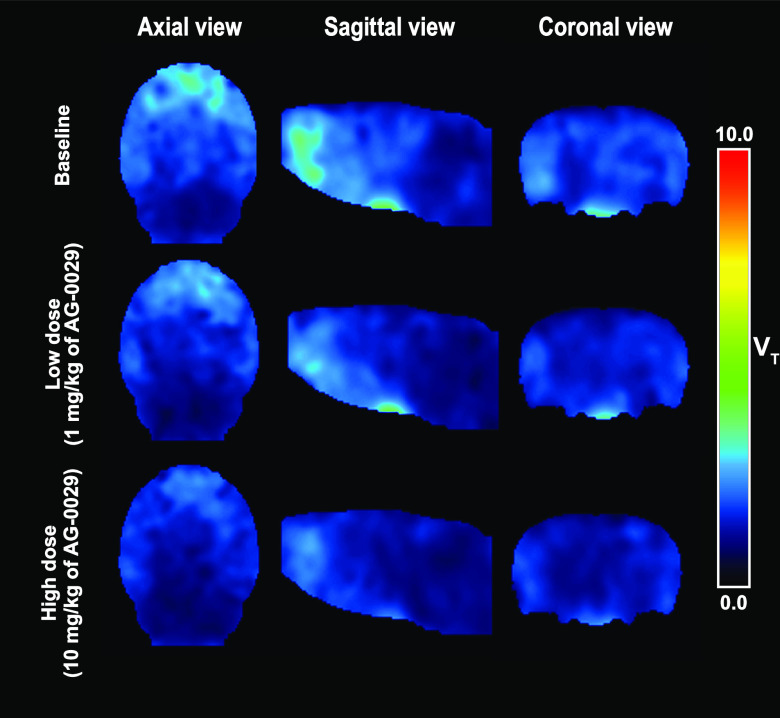
Parametric (*V*_T_) images of [^11^C]GSK-189254 binding (axial, coronal, and sagittal views) of a representative rat brain at baseline (top row) and after administration of 1 mg/kg (middle row), or 10 mg/kg of AG-0029 (bottom row).

Parametric (*V*_T_) images of [^11^C]GSK-189254 binding in rat brain at baseline and after administration of 1 and 10 mg/kg doses of AG-0029 are shown in Figure 9. The first row of Figure 9 shows the *V*_T_ values derived from Logan graphical analysis at baseline which demonstrates a clear binding of the tracer in the frontal lobe including the frontal cortex, striatum, and parietal cortex with high expression of H_3_ receptors.^55^ The second and third rows display the images of rats that received a low (1 mg/kg) or high (10 mg/kg) dose of AG-0029 drug, resulting in reduced *V*_T_ values throughout the brain.

**Figure 10:**
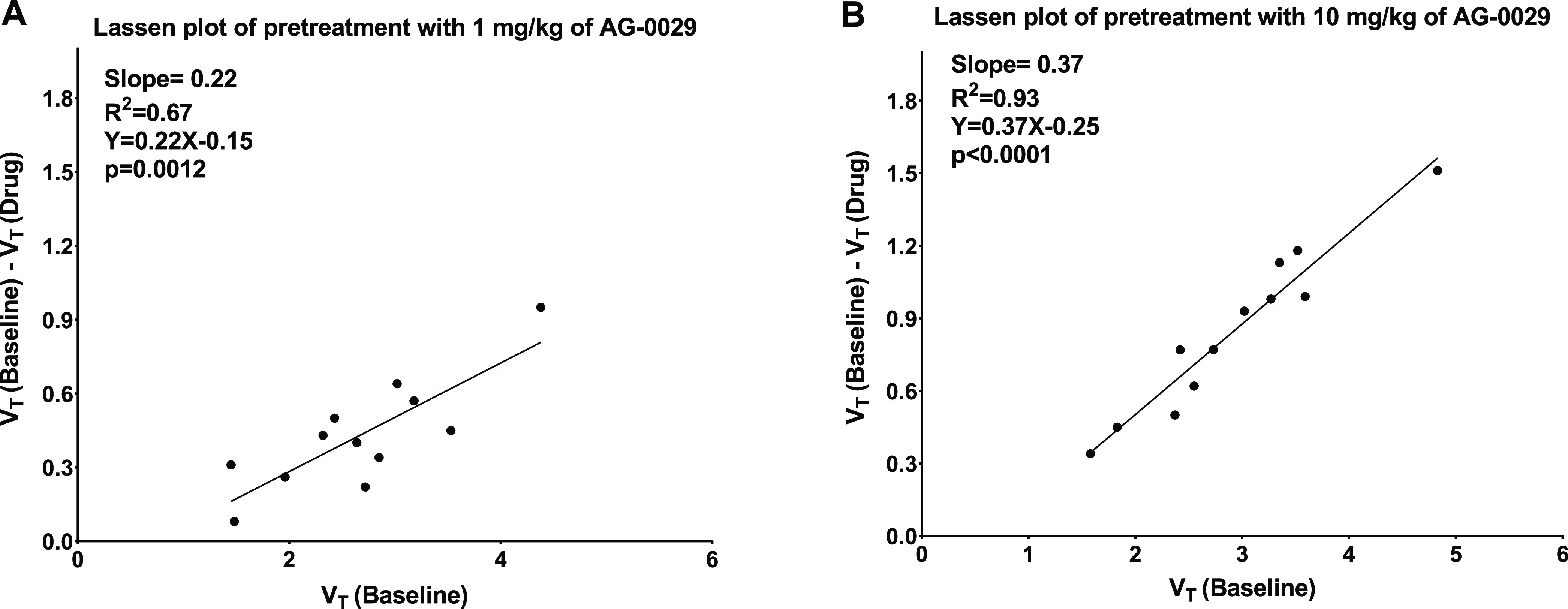
Examples of Lassen plots from (A) a rat that received a low dose (1 mg/kg) of AG-0029 and (B) a rat that received a high dose (10 mg/kg) of the drug before the post-dose [^11^C]GSK-189254 PET scan.

The occupancy values of AG-0029 in the brain of rats were derived from the Lassen plot because binding potentials of [^11^C]GSK-189254 cannot be reliably estimated and a suitable reference region is not available. Figure 10 shows examples of the Lassen plot for the two doses of the administrated drug. In general, the Lassen plot showed a better fit for the high-dose (*R*^2^ 0.93) than for the low-dose (*R*^2^ 0.67) data. The measured receptor occupancies, as determined from the slope of the Lassen plot, for the two administered doses of AG-0029 were 11.9 ± 8.5% (1 mg/kg) and 40.3 ± 11.3% (10 mg/kg).

## Discussion

This study aimed to demonstrate the in vivo engagement of the anti-Parkinsonian dual-action drug AG-0029 to dopamine D_2/_D_3_ and histamine H_3_ receptors in the brain of healthy rats by PET imaging, using the D_2_/D_3_ receptor ligand, [^11^C]raclopride, and the H_3_ receptor ligand, [^11^C]GSK-189254. In functional tests, AG-0029 was identified as a very potent dopamine D_2_/D_3_ receptor agonist (EC_50_ 0.08 nM) and a moderately potent H_3_ receptor antagonist (IC_50_ 111 nM).^39^ Our PET data showed that AG-0029 can rapidly cross the blood–brain barrier and interact with D_2_/D_3_ and H_3_ receptors (Figures 6 and 9). The results of our in vivo imaging study are in line with the outcome of tests performed previously by the manufacturer. We found that administration of AG-0029 results in a high occupancy of the striatal dopamine D_2_/D_3_ receptors (84% at the dose of 1 mg/kg) and a moderate occupancy of the histamine H_3_ receptors (40% at a dose of 10 mg/kg) in the brain (Figures 5 and 10).

There are not many records of PET occupancy studies for agonist drugs targeting D_2/3_ receptors since an agonist could have strong physiological effects, such as widening of blood vessels, alteration of the heart rate, or a drop in the blood pressure.^56−58^ Another complication of studying the occupancy of an agonist is that it binds preferably to the high-affinity state of the G-protein-coupled receptors, which may be a small fraction of the total receptor population and therefore be difficult to measure. However, when administrated at a high dose, an agonist drug will also occupy the receptor population in the low-affinity state. Competition with the endogenous ligand (dopamine) might also complicate the study. Despite these potential complications, we were able to demonstrate a high receptor occupancy of the striatal D_2_/D_3_ receptor in the rat brain by the agonist AG-0029, using [^11^C]raclopride PET.^59^ In a previous study from our institution, the occupancy of the D_2/_D_3_ agonist (+)-PD 128907 was measured over a dose range from 10 to 10 000 nmol/kg in *Macaca mulatta* males with [^11^C]raclopride PET.^59^ The receptor occupancy by (+)-PD 128907 increased in an orderly dose-dependent manner to a maximum of 85%, which is near our estimated occupancy for AG-0029.^59^ A similar dose-dependent occupancy of D_2_/D_3_ receptors by an agonist has been reported for the clinically applied drug ropinirole. In a PET study with the radioligand [^18^F]fallypride, receptor occupancies of 9.6 and 56% were measured in the rat brain after administration of 5 and 15 mg/kg ropinirole, respectively.^60^ To achieve considerable D_2_/D_3_ receptor occupancy, more than a 20-fold higher dose of ropinirole was required than was needed in our study for AG-0029. This difference may be related to the much lower affinity (EC_50_) of ropinrole (970 nM) to D_2_/D_3_ receptors in comparison with AG-0029 (0.08 nM). Yet, these studies all show the feasibility of assessing the receptor occupancy of an agonist for the D_2_/D_3_ receptor with [^11^C]raclopride PET.

For the assessment of the binding of AG-0029 as an antagonist to the H_3_-receptor, PET imaging with [^11^C]GSK-189254 was used. So far, this tracer has not been used in occupancy studies in rodents. A high dose of AG-0029 reduced the uptake of [^11^C]GSK-189254 in brain regions with high H_3_-receptor expression in healthy rats, but a low dose of AG-0029 increased these. In regions with low receptor expression, such as the cerebellum, administration of both doses of AG-0029 resulted in enhanced tracer uptake. These increments in tracer uptake might originate from the fact that the SUV measurements cannot distinguish specific from nonspecific uptake. SUV is sensitive to various confounding factors like nonspecific binding, plasma clearance, metabolism, and perfusion.^61^ Metabolite analysis indicated that tracer metabolism was not substantially affected by the administration of the test drug (Figure 7C). However, tracer concentrations in blood and plasma were increased after administration of either concentration of AG-0029 (Figure 7A,B). Even though this effect was statistically not significant, this might suggest that increased tracer delivery to the brain after the administration of AG-0029 may have been responsible for the observed increase in brain uptake.

AG-0029 may affect tracer clearance by competing with the tracer for drug transporters in liver or kidney, or for a drug-metabolizing enzyme such as cytochrome P450. Due to such competition, the tracer is less rapidly cleared from the blood and more tracer is delivered to the brain. However, the interaction of drug and tracer seems complex, since an increase of SUV values in the brain TACs was observed only at the 1 mg/kg dose of AG-0029 and not at the 10 mg/kg dose. In addition to affecting tracer transport and metabolism in excretory organs, AG-0029 also affected heart rate and cardiac output (Figures 2 and 4). Moreover, AG-0029 could dilate (or constrict) blood vessels and could thus affect tracer delivery to the brain. Irrespective of the cause, these results show that quantification of [^11^C]GSK-189254 PET data through pharmacokinetic modeling is crucial for adequate data analysis.

For D_2_/D_3_ receptor imaging with the ligand [^11^C]raclopride, a reference region devoid of target receptors is available and consequently, BP_ND_ values can be estimated with a reference tissue model.^62,63^ For imaging of H_3_-receptors with [^11^C]GSK-189254, no suitable reference tissue is available and estimation of BP_ND_ values by compartment modeling with a plasma input function gives unreliable results. Reliable estimation of the *V*_T_ of [^11^C]GSK-189254 is feasible,^54^ but the *V*_T_ of a single target region is not suitable to assess receptor occupancy, since *V*_T_ contains not only a specific binding component (*V*_S_) but also a nondisplaceable (*V*_ND_) component, which cannot be easily assessed without a reference tissue. To overcome this hurdle, the target engagement (drug occupancy) for each subject in the study can be determined with the Lassen plot by assuming that *V*_ND_ and the receptor occupancy are the same in all brain regions, whereas the specific binding of the tracer (receptor density) is different in different regions.^49,64^ In a Lassen plot, the difference of *V*_T_ at baseline and *V*_T_ post-dose for each brain region is plotted against *V*_T_ at baseline for that region. In general, the Lassen plot of our [^11^C]GSK-189254 data provided better fits for the high dose (10 mg/kg) than for the low dose (1 mg/kg) of AG-0029. This could be due to the fact that for the lower dose, the difference in specific binding between the baseline and post-dose scans is relatively small and possibly in the same order of magnitude as the uncertainties in the estimations of the *V*_T_ values. This would result in a relatively high error in the estimated receptor occupancy. At higher doses, differences in *V*_T_ are much larger, which would result in a more reliable estimation of the receptor occupancy.

In a PET study with [^11^C]TASP-0410457 in rhesus monkeys, a dose of 3 mg/kg of the antagonist ciproxifan resulted in an H_3_ receptor occupancy of 75%, based on a Lassen plot.^65^ The discrepancy between this monkey study and our PET study in rodents may be related to the fact that ciproxifan has a higher affinity to H_3_ receptors than AG-0029 (IC_50_ values 40 and 111 nM, respectively).^66^ Another explanation could be the existence of species differences in receptor expression, affinity, or other physiological parameters between monkeys and rats.^67^ In a clinical PET study with [^11^C]GSK-189254, a therapeutic dose (40 mg daily) of pitolisant (inverse agonist/antagonist) was associated with an H_3_ receptor occupancy of 84%,^68^ which suggests that the occupancy of AG-0029 observed in our study may be too low for therapeutic efficacy. Since our [^11^C]GSK-189254 data indicated moderate H_3_ receptor occupancy even at the 10 mg/kg dose, higher drug doses (30 or 50 mg/kg) or multiple doses of AG-0029 seem to be required to achieve therapeutic effects of H3 receptor blockade. Such doses would result in virtually complete occupancy of the D2 receptor population with the possibility of undesired adverse side effects.

## Conclusions

Dopamine D_2_/D_3_ receptor occupancy by AG-0029 in the striatum of rats could be reliably estimated from the [^11^C]raclopride BP_ND_, resulting in an occupancy of 84% at a dose of 1 mg/kg. H_3_ receptor occupancy by AG-0029 can be estimated using [^11^C]GSK-189254 and a Lassen plot, which indicated 12 and 40% H_3_ receptor occupancies at doses of 1 and 10 mg/kg, respectively. For substantial occupancy of H_3_ receptor and therapeutic effect of H_3_ receptor blockade, a much (10- to 30-fold) higher dose of AG-0029 appears to be required than for occupancy of the D_2_/D_3_ receptor population. A successful dual-action drug for Parkinson’s disease may need to have D_2_ and H_3_ receptor affinities in a similar range, not as widely apart as AG-0029.

## References

[ref1] Nussbaum R. L.; Ellis C. E. Alzheimer’s disease and Parkinson’s disease. N. Engl. J. Med. 2003, 348, 1356–1364. 10.1056/NEJM2003ra020003. 12672864

[ref2] Schulz-Schaeffer W. J. The synaptic pathology of α-synuclein aggregation in dementia with Lewy bodies, Parkinson’s disease and Parkinson’s disease dementia. Acta Neuropathol. 2010, 120, 131–143. 10.1007/s00401-010-0711-0. 20563819 PMC2892607

[ref3] Villar-Piqué A.; Lopes da Fonseca T.; Outeiro T. F. Structure, function and toxicity of alpha-synuclein: the Bermuda triangle in synucleinopathies. J. Neurochem. 2016, 139, 240–255. 10.1111/jnc.13249. 26190401

[ref4] Burré J.; Sharma M.; Südhof T. C. Cell biology and pathophysiology of α-synuclein. Cold Spring Harbor Perspect. Med. 2018, 8, a024091 10.1101/cshperspect.a024091. PMC5519445 28108534

[ref5] Kalia L. V.; Lang A. E. Parkinson’s disease. Lancet 2015, 386, 896–912. 10.1016/S0140-6736(14)61393-3. 25904081

[ref6] Schlicker E.; Malinowska B.; Kathmann M.; Göthert M. Modulation of neurotransmitter release via histamine H3 heteroreceptors. Fundam. Clin. Pharmacol. 1994, 8, 128–137. 10.1111/j.1472-8206.1994.tb00789.x. 8020871

[ref7] Blandina P.; Bacciottini L.; Giovannini M. H3 Receptor Modulation of the Release of Neurotransmitters In Vivo. In Pharmacochemistry Library., Elsevier, 1998; pp 27–40.

[ref8] Kaasinen V.; Ruottinen H. M.; Någren K.; et al. Upregulation of putaminal dopamine D2 receptors in early Parkinson’s disease: a comparative PET study with [11C] raclopride and [11C] N-methylspiperone. J. Nucl. Med. 2000, 41, 65–70. 10647606

[ref9] Hwang W-J.; Yao W-J.; Wey S-P.; et al. Downregulation of striatal dopamine D2 receptors in advanced Parkinson’s disease contributes to the development of motor fluctuation. Eur. Neurol. 2002, 47, 113–117. 10.1159/000047962. 11844900

[ref10] Kaasinen V.; Vahlberg T.; Stoessl A. J.; et al. Dopamine Receptors in Parkinson’s Disease: A Meta-Analysis of Imaging Studies. Mov. Disord. 2021, 36, 1781–1791. 10.1002/mds.28632. 33955044

[ref11] Bergman H.; Deuschl G. Pathophysiology of Parkinson’s disease: from clinical neurology to basic neuroscience and back. Mov. Disord. 2002, 17, S28–S40. 10.1002/mds.10140. 11948753

[ref12] Davie C. A. A review of Parkinson’s disease. Br. Med. Bull. 2008, 86, 109–127. 10.1093/bmb/ldn013. 18398010

[ref13] Łażewska D.; Olejarz-Maciej A.; Reiner D.; et al. Dual target ligands with 4-tert-butylphenoxy scaffold as histamine H3 receptor antagonists and monoamine oxidase B inhibitors. Int. J. Mol. Sci. 2020, 21, 3411 10.3390/ijms21103411. PMC7279487 32408504

[ref14] Sharma A.; Muresanu D. F.; Patnaik R.; et al. Histamine H3 and H4 receptors modulate Parkinson’s disease induced brain pathology. Neuroprotective effects of nanowired BF-2649 and clobenpropit with anti-histamine-antibody therapy. Prog. Brain Res. 2021, 266, 1–73. 10.1016/bs.pbr.2021.06.003. 34689857

[ref15] Oertel W.; Schulz J. B. Current and experimental treatments of Parkinson disease: A guide for neuroscientists. J. Neurochem. 2016, 139, 325–337. 10.1111/jnc.13750. 27577098

[ref16] Jaiteh M.; Zeifman A.; Saarinen M.; et al. Docking screens for dual inhibitors of disparate drug targets for Parkinson’s disease. J. Med. Chem. 2018, 61, 5269–5278. 10.1021/acs.jmedchem.8b00204. 29792714 PMC6716773

[ref17] Ntetsika T.; Papathoma P-E.; Markaki I. Novel targeted therapies for Parkinson’s disease. Mol. Med. 2021, 27, 17 10.1186/s10020-021-00279-2. 33632120 PMC7905684

[ref18] Stocchi F.; Antonini A.; Berg D.; et al. Safinamide in the treatment pathway of Parkinson’s Disease: a European Delphi Consensus. npj Parkinson’s Dis. 2022, 8, 17 10.1038/s41531-022-00277-z. 35190544 PMC8861053

[ref19] Clapham J.; Kilpatrick G. Histamine H3 receptors modulate the release of [3H]-acetylcholine from slices of rat entorhinal cortex: evidence for the possible existence of H3 receptor subtypes. Br. J. Pharmacol. 1992, 107, 919 10.1111/j.1476-5381.1992.tb13386.x. 1334753 PMC1907926

[ref20] Anichtchik O. V.; Peitsaro N.; Rinne J. O.; et al. Distribution and modulation of histamine H3 receptors in basal ganglia and frontal cortex of healthy controls and patients with Parkinson’s disease. Neurobiol. Dis. 2001, 8, 707–716. 10.1006/nbdi.2001.0413. 11493035

[ref21] Molina-Hernández A.; Nuñez A.; Sierra J-J.; Arias-Montaño J. A. Histamine H3 receptor activation inhibits glutamate release from rat striatal synaptosomes. Neuropharmacology 2001, 41, 928–934. 10.1016/S0028-3908(01)00144-7. 11747897

[ref22] Osorio-Espinoza A.; Alatorre A.; Ramos-Jimenez J.; et al. Pre-synaptic histamine H3 receptors modulate glutamatergic transmission in rat globus pallidus. Neuroscience 2011, 176, 20–31. 10.1016/j.neuroscience.2010.12.051. 21195747

[ref23] Nowak P.; Bortel A.; Dabrowska J.; et al. Histamine H3 receptor ligands modulate L-dopa-evoked behavioral responses and L-dopa-derived extracellular dopamine in dopamine-denervated rat striatum. Neurotoxicity Res. 2008, 13, 231–240. 10.1007/BF03033506. 18522902

[ref24] Schwartz J. C. The histamine H3 receptor: from discovery to clinical trials with pitolisant. Br. J. Pharmacol. 2011, 163, 713–721. 10.1111/j.1476-5381.2011.01286.x. 21615387 PMC3111674

[ref25] Hancock A. A.; Fox G. B. Perspectives on cognitive domains, H3 receptor ligands and neurological disease. Expert Opin. Invest. Drugs 2004, 13, 1237–1248. 10.1517/13543784.13.10.1237. 15461554

[ref26] Medhurst A. D.; Atkins A. R.; Beresford I. J.; et al. GSK189254, a novel H3 receptor antagonist that binds to histamine H3 receptors in Alzheimer’s disease brain and improves cognitive performance in preclinical models. J. Pharmacol. Exp. Ther. 2007, 321, 1032–1045. 10.1124/jpet.107.120311. 17327487

[ref27] Esbenshade T. A.; Browman K.; Bitner R.; et al. The histamine H3 receptor: an attractive target for the treatment of cognitive disorders. Br. J. Pharmacol. 2008, 154, 1166–1181. 10.1038/bjp.2008.147. 18469850 PMC2483387

[ref28] Stocking E. M.; Letavic M. A. Histamine H3 antagonists as wake-promoting and pro-cognitive agents. Curr. Top. Med. Chem. 2008, 8, 988–1002. 10.2174/156802608784936728. 18673168

[ref29] Chazot P. L. Therapeutic potential of histamine H3 receptor antagonists in dementias. Drug News Perspect. 2010, 23, 99–103. 10.1358/dnp.2010.23.2.1475899. 20369074

[ref30] Brioni J. D.; Esbenshade T. A.; Garrison T. R.; et al. Discovery of histamine H3 antagonists for the treatment of cognitive disorders and Alzheimer’s disease. J. Pharmacol. Exp. Ther. 2011, 336, 38–46. 10.1124/jpet.110.166876. 20864505

[ref31] Griebel G.; Pichat P.; Pruniaux M-P.; et al. SAR110894, a potent histamine H3-receptor antagonist, displays procognitive effects in rodents. Pharmacol. Biochem. Behav. 2012, 102, 203–214. 10.1016/j.pbb.2012.04.004. 22542742

[ref32] Vohora D.; Bhowmik M. Histamine H3 receptor antagonists/inverse agonists on cognitive and motor processes: relevance to Alzheimer’s disease, ADHD, schizophrenia, and drug abuse. Front. Syst. Neurosci. 2012, 6, 72 10.3389/fnsys.2012.00072. 23109919 PMC3478588

[ref33] Nikolic K.; Filipic S.; Agbaba D.; Stark H. Procognitive properties of drugs with single and multitargeting H3 receptor antagonist activities. CNS Neurosci. Ther. 2014, 20, 613–623. 10.1111/cns.12279. 24836924 PMC6493064

[ref34] Sadek B.; Saad A.; Sadeq A.; et al. Histamine H3 receptor as a potential target for cognitive symptoms in neuropsychiatric diseases. Behav. Brain Res. 2016, 312, 415–430. 10.1016/j.bbr.2016.06.051. 27363923

[ref35] Masini D.; Lopes-Aguiar C.; Bonito-Oliva A.; et al. The histamine H3 receptor antagonist thioperamide rescues circadian rhythm and memory function in experimental parkinsonism. Transl. Psychiatry 2017, 7, e1088 10.1038/tp.2017.58. 28398338 PMC5416699

[ref36] Aarsland D.; Andersen K.; Larsen J. P.; et al. Prevalence and characteristics of dementia in Parkinson disease: an 8-year prospective study. Arch. Neurol. 2003, 60, 387–392. 10.1001/archneur.60.3.387. 12633150

[ref37] Svenningsson P.; Westman E.; Ballard C.; Aarsland D. Cognitive impairment in patients with Parkinson’s disease: diagnosis, biomarkers, and treatment. Lancet Neurol. 2012, 11, 697–707. 10.1016/S1474-4422(12)70152-7. 22814541

[ref38] Sasikumar S.; Strafella A. P. Imaging mild cognitive impairment and dementia in Parkinson’s disease. Front. Neurol. 2020, 11, 47 10.3389/fneur.2020.00047. 32082250 PMC7005138

[ref39] Heeres A.; Willigers-Hogg S.; Borst M. L. G.; Joyce-Droge M. Multiple D2 a(nta)gonist/H3 antagonist for treatment of CNS related disorders. Patent WO2015069110, 2015.

[ref40] Heeres M. D. A.; Andrew M.; Isaac V.; Sandra H. Design and Synthesis of Multiple Ligands for the Treatment of Motor and Cognitive Symptoms of Parkinson’s Disease. In MCB2014: Joining Forces in Pharmaceutical Analysis and Medicinal Chemistry; University of Groningen: Groningen, 2014; p 24.

[ref41] Caruana E. J.; Roman M.; Hernández-Sánchez J.; et al. Longitudinal studies. J. Thoracic Dis. 2015, 7, E537 10.3978/j.issn.2072-1439.2015.10.63. PMC4669300 26716051

[ref42] Langer O.; Någren K.; Dolle F.; et al. Precursor synthesis and radiolabelling of the dopamine D2 receptor ligand [11C] raclopride from [11C] methyl triflate. J. Labelled Compd. Radiopharm. 1999, 42, 1183–1193. 10.1002/(SICI)1099-1344(199912)42:12<1183::AID-JLCR274>3.0.CO;2-Z.

[ref43] Plisson C.; Gunn R. N.; Cunningham V. J.; et al. 11C-GSK189254: a selective radioligand for in vivo central nervous system imaging of histamine H3 receptors by PET. J. Nucl. Med. 2009, 50, 2064–2072. 10.2967/jnumed.109.062919. 19910432

[ref44] Wang M.; Gao M.; Steele B. L.; et al. A new facile synthetic route to [11C] GSK189254, a selective PET radioligand for imaging of CNS histamine H3 receptor. Bioorg. Med. Chem. Lett. 2012, 22, 4713–4718. 10.1016/j.bmcl.2012.05.076. 22687746

[ref45] Sijbesma J. W. A.; Zhou X.; García D. V.; et al. Novel approach to repeated arterial blood sampling in small animal PET: application in a test-retest study with the adenosine A1 receptor ligand [11 C] MPDX. Mol. Imaging Biol. 2016, 18, 715–723. 10.1007/s11307-016-0954-9. 27091332 PMC5010612

[ref46] Garcia D. V.; Casteels C.; Schwarz A. J.; et al. A standardized method for the construction of tracer specific PET and SPECT rat brain templates: validation and implementation of a toolbox. PLoS One 2015, 10, e0143900 10.1371/journal.pone.0143900. 25823005 PMC4379068

[ref47] Wu Y.; Carson R. E. Noise reduction in the simplified reference tissue model for neuroreceptor functional imaging. J. Cerebral Blood Flow Metab. 2002, 22, 1440–1452. 10.1097/01.WCB.0000033967.83623.34. 12468889

[ref48] Ghazanfari N.; van Waarde A.; Doorduin J.; Sijbesma J. W. A.; Kominia M.; Koelewijn M.; Attia K.; Willemsen A. T. M.; Visser T. J.; Heeres A.; Dierckx R. A. J. O.; de Vries E. F. J.; Elsinga P. H. Pharmacokinetic Modeling of [11C]GSK-189254 PET tracer targeting H3 receptors in Rat Brain. Mol. Pharmaceutics 2022, 19, 918–928. 10.1021/acs.molpharmaceut.1c00889. PMC8905578 35170965

[ref49] Cunningham V. J.; Rabiner E. A.; Slifstein M.; et al. Measuring drug occupancy in the absence of a reference region: the Lassen plot re-visited. J. Cerebral Blood Flow Metab. 2010, 30, 46–50. 10.1038/jcbfm.2009.190. PMC2949110 19738632

[ref50] Hardin J. W. Generalized Estimating Equations (GEE). In Encyclopedia of Statistics in Behavioral Science, Chapman and Hall/CRC, 2005.

[ref51] Zhang Y.; Fox G. B. PET imaging for receptor occupancy: meditations on calculation and simplification. J. Biomed. Res. 2012, 26, 69–76. 10.1016/S1674-8301(12)60014-1. 23554733 PMC3597321

[ref52] van Waarde A. Measuring receptor occupancy with PET. Curr. Pharm. Des. 2000, 6, 1593–1610. 10.2174/1381612003398951. 10974155

[ref53] Cheng Y.-C.; Prusoff W. H. Relationship between the inhibition constant (Ki) and the concentration of inhibitor which causes 50 per cent inhibition (I50) of an enzymatic reaction. Biochem. Pharmacol. 1973, 22, 3099–3108. 10.1016/0006-2952(73)90196-2. 4202581

[ref54] Ghazanfari N.; van Waarde A.; Doorduin J.; et al. Pharmacokinetic Modeling of [11C] GSK-189254, PET Tracer Targeting H3 Receptors, in Rat Brain. Mol. Pharmaceutics 2022, 19, 918–928. 10.1021/acs.molpharmaceut.1c00889. PMC8905578 35170965

[ref55] Pillot C.; Héron A.; Cochois V.; et al. A detailed mapping of the histamine H3 receptor and its gene transcripts in rat brain. Neuroscience 2002, 114, 173–193. 10.1016/S0306-4522(02)00135-5. 12207964

[ref56] Polakowski J. S.; Segreti J. A.; Cox B. F.; et al. Effects of selective dopamine receptor subtype agonists on cardiac contractility and regional haemodynamics in rats. Clin. Exp. Pharmacol. Physiol. 2004, 31, 837–841. 10.1111/j.1440-1681.2004.04095.x. 15659045

[ref57] Farha K. A.; Baljé-Volkers C.; Tamminga W.; et al. Dopamine D2R agonist-induced cardiovascular effects in healthy male subjects: potential implications in clinical settings. Int. Scholarly Res. Not. 2014, 2014, 956353 10.1155/2014/956353. PMC3920609 24587918

[ref58] Zhang P.; Li Y.; Nie K.; et al. Hypotension and bradycardia, a serious adverse effect of piribedil, a case report and literature review. BMC Neurol. 2018, 18, 221 10.1186/s12883-018-1230-1. 30591018 PMC6307137

[ref59] Kortekaas R.; Maguire R. P.; Cremers T. I.; et al. In vivo Binding Behavior of Dopamine Receptor Agonist (+)– PD 128907 and Implications for the “Ceiling Effect” in Endogenous Competition Studies with [11C] Raclopride—a Positron Emission Tomography Study in Macaca mulatta. J. Cerebral Blood Flow Metab. 2004, 24, 531–535. 10.1097/00004647-200405000-00007. 15129185

[ref60] Huang C.; Wang Z.; Liu L.; et al. Predicting the dopamine D2 receptor occupancy of ropinirole in rats using positron emission tomography and pharmacokinetic–pharmacodynamic modeling. Xenobiotica 2019, 49, 143–151. 10.1080/00498254.2018.1428383. 29334326

[ref61] Yoder K. K.; Territo P. R.; Hutchins G. D.; et al. Comparison of standardized uptake values with volume of distribution for quantitation of [11C] PBR28 brain uptake. Nucl. Med. Biol. 2015, 42, 305–308. 10.1016/j.nucmedbio.2014.11.003. 25487553 PMC4329090

[ref62] Lammertsma A. A.; Hume S. P. Simplified reference tissue model for PET receptor studies. NeuroImage 1996, 4, 153–158. 10.1006/nimg.1996.0066. 9345505

[ref63] Lammertsma A. A.; Bench C.; Hume S.; et al. Comparison of methods for analysis of clinical [11C] raclopride studies. J. Cerebral Blood Flow Metab. 1996, 16, 42–52. 10.1097/00004647-199601000-00005. 8530554

[ref64] Lassen N. A.; Bartenstein P.; Lammertsma A.; et al. Benzodiazepine receptor quantification in vivo in humans using [11C] flumazenil and PET: application of the steady-state principle. J. Cerebral Blood Flow Metab. 1995, 15, 152–165. 10.1038/jcbfm.1995.17. 7798333

[ref65] Koga K.; Maeda J.; Tokunaga M.; et al. Development of TASP0410457 (TASP457), a novel dihydroquinolinone derivative as a PET radioligand for central histamine H 3 receptors. EJNMMI Res. 2016, 6, 11 10.1186/s13550-016-0170-2. 26860293 PMC4747952

[ref66] Hagenow S.; Stasiak A.; Ramsay R.; Stark H. Ciproxifan, a histamine H 3 receptor antagonist, reversibly inhibits monoamine oxidase A and B. Sci. Rep. 2017, 7, 40541 10.1038/srep40541. 28084411 PMC5233962

[ref67] Lovenberg T. W.; Pyati J.; Chang H.; et al. Cloning of rat histamine H3 receptor reveals distinct species pharmacological profiles. J. Pharmacol. Exp. Ther. 2000, 293, 771–778. 10869375

[ref68] Rusjan P.; Sabioni P.; Di Ciano P.; et al. Exploring occupancy of the histamine H3 receptor by pitolisant in humans using PET. Br. J. Pharmacol. 2020, 177, 3464–3472. 10.1111/bph.15067. 32293706 PMC7348085

